# From choice architecture to choice engineering

**DOI:** 10.1038/s41467-019-10825-6

**Published:** 2019-06-26

**Authors:** Ohad Dan, Yonatan Loewenstein

**Affiliations:** 1 Department of Cognitive Sciences, The Hebrew University, Jerusalem, Israel; 2 The Federmann Center for the Study of Rationality, The Hebrew University, Jerusalem, Israel; 3 The Alexander Silberman Institute of Life Sciences, The Hebrew University, Jerusalem, Israel; 4 The Edmond and Lily Safra Center for Brain Sciences, The Hebrew University, Jerusalem, Israel

**Keywords:** Cognitive neuroscience, Computational neuroscience, Learning and memory, Reward, Human behaviour

## Abstract

Qualitative psychological principles are commonly utilized to influence the choices that people make. Can this goal be achieved more efficiently by using quantitative models of choice? Here, we launch an academic competition to compare the effectiveness of these two approaches.

Influencing human choices has been a principal objective of parents and educators, as well as of salesmen and politicians for millennia. In economics, psychology and neuroscience, there is considerable interest in the principles underlying decision-making and in the ways in which they can be used to bias human choice. The 2017 Nobel prize in economics, was awarded to Richard Thaler for his contributions to the development of behavioral economics and its applications to policy-making. Thaler coined the term choice architecture to describe how insights from behavioral economics can be used to nudge choices without changing their objective values^1^. Choice architecture utilizes qualitative psychological principles to shape behavior. Can this goal be more effectively achieved using quantitative models? In the natural sciences, quantitative models underlay the development of engineering. Therefore, we ask whether quantitative models can revolutionize the field of choice architecture into choice engineering, defined as the use of quantitative models to shape choice behavior.

Both qualitative principles and quantitative models identify factors that affect behavior. The difference between them is that the latter, but not the former, quantitatively describe the magnitudes of the effects. Operant learning is a process, in which the strength or likelihood of a behavior is modified by rewards and punishments. We demonstrate the difference between choice architecture and choice engineering in the framework of an operant learning task.

Consider an objective function of maximally biasing choices in favor of a predefined alternative (defined here as alternative 1) in a repeated, two-alternative forced-choice task with binary rewards (Fig. 1a). How should a choice architect and a choice engineer allocate the rewards in view of this goal? Thorndike’s Law of Effect is a qualitative description of operant learning: “Of several responses made to the same situation, those which are accompanied or closely followed by satisfaction to the animal … will be more likely to recur”^2^. In line with this law of behavior, a choice architect (and common-sense intuition) would recommend allocating all available rewards to the desired alternative 1 (Fig. 1b). Subtler principles of choice become necessary if we add constraints, e.g., that the number of rewards that can be allocated to alternative 1 is limited. For a real-life example, assume that you organize a seminar course, and invite each week a different guest lecturer. Having taught the course in the past you know in advance, which lectures will be more interesting. How should you distribute those more interesting lecturers between the different weeks, if your goal is to maximize students’ attendance throughout the year?

**Fig. 1:**
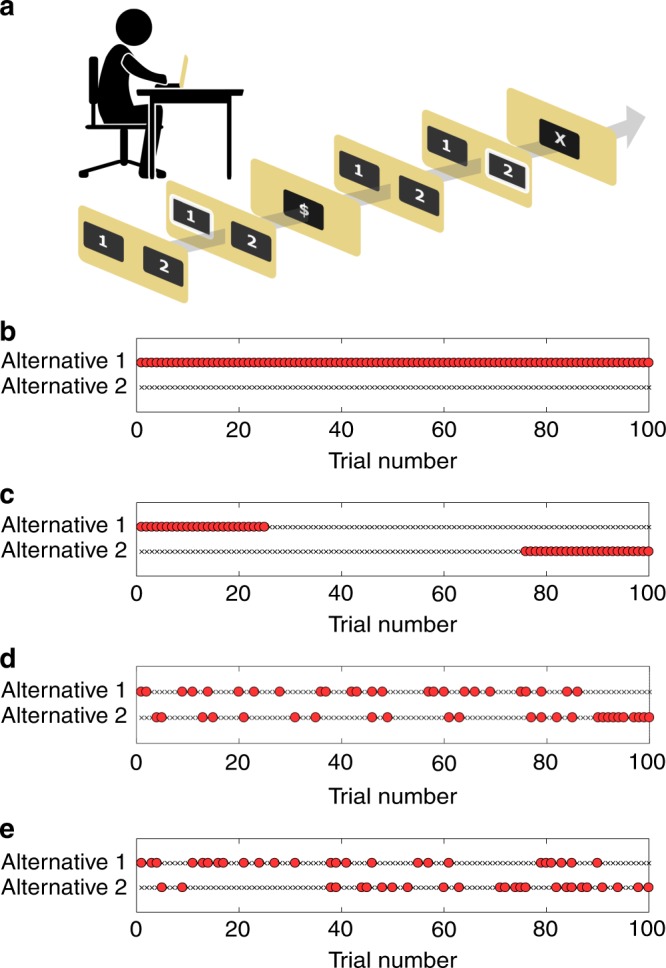
Choice engineering in a repeated two-alternative forced-choice task. **a** Experimental task—A reward schedule allocates binary rewards to each of the alternatives in each of the trials. The subject repeatedly chooses between the two alternatives. If the subject chooses a rewarded alternative, then she receives a monetary reward in that trial. In this example, the first choice is “1” and it yields a reward ($ sign) while the second choice, “2”, does not yield any reward. No feedback is given about the foregone payoff (the reward that was associated with the alternative that was not chosen). **b** Based on the Law of Effect, bias in favor of alternative 1 is expected to be maximal if all choices of alternative 1 are associated with a reward (red circles) while choosing alternative 2 is never rewarded (black X). **c** Choice architecture. If the number of rewards associated with the two alternatives is constrained, a choice-architect may choose to use the primacy heuristic and place all rewards associated with alternative 1 at the beginning of the sequence and those of alternative 2 at its end. **d**, **e** A choice engineer can utilize a quantitative model of choice to optimize the reward schedule. **d** Static schedule optimized for a QL agent. **e** Static schedule optimized for a CATIE agent

For simplicity, we consider a symmetric problem in which an identical predefined number of rewards must be assigned to each alternative. The Law of Effect is not specific enough to prescribe the optimal allocation of rewards in this case. However, a choice architect may utilize additional qualitative psychological principle to allocate the rewards. For example, motivated by the primacy effect^3^ the architect may recommend to place all rewards that are associated with alternative 1 at the beginning of the sequence and those of alternative 2 at its end (Fig. 1c).

Quantitative models are widely used to study the computational principles and neural basis underlying operant learning^4,5^. For example, the popular Q-learning (QL) model^6^ provides a quantitative description of learning in the repeated, two-alternative forced-choice task. According to this model, humans compute the expected reward associated with each of the alternatives based on their past experience, and choose more often the alternative associated with the higher estimated expected reward. The QL model provides a quantitative prediction of future choices—the probability that the participant would choose each of the alternatives in each trial, based on her specific history of actions and their outcomes (see Supplementary Methods). A choice engineer that is equipped with such an accurate quantitative description of the learning process can design a more sophisticated reward schedule for this task (Fig. 1d, e).

## The choice engineering competition

Are current models of choice accurate enough to engineer behavior? To address this question, we follow the grand tradition of competitions in neuroscience, cognitive sciences and game-theory^7–9^. In recent years, academic competitions proved to be a major catalyst in the field of computer science. Most notably, the ImageNet^10^ challenge, served as a prominent driver of both theoretical and practical advancements in the field of computer vision and Deep Learning.

Here we announce the Choice Engineering Competition. The challenge presented to the researchers participating in our competition is to propose a reward schedule that maximally biases the choices of human subjects in a repeated, two-alternative, forced-choice experiment, which we denote as a session. A session consists of 100 trials. In each trial, a reward may be assigned to one, two or none of the alternatives, complying with the global constraint of assigning a reward to exactly one-quarter of the trials (25 trials) of each alternative (as in Fig. 1c–e).

## Static reward schedule

**Fig. 2:**
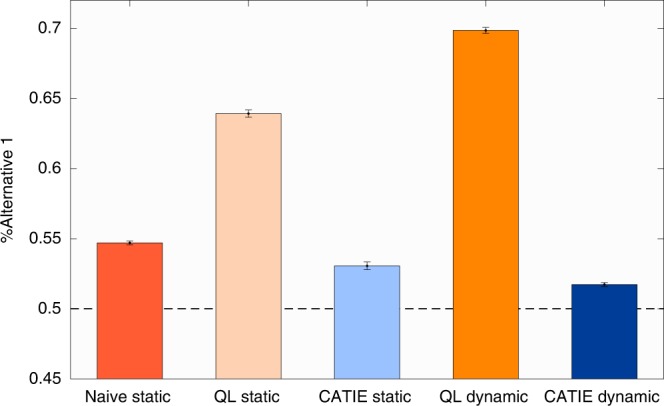
The effect of reward schedule on the bias of a QL agent. Red, orange and blue bars denote the naïve schedule, and schedules optimized assuming a QL agent and a CATIE agent, respectively. Light colors denote static schedules whereas dark colors denote a dynamic reward schedule (see Supplementary Methods). Dashed line denotes chance level and error-bars are standard errors of the mean

One way of complying with these constraints is to prepare, in advance, the allocation of rewards to trials (as in Fig. 1c–e). We refer to such schedules as static. There are approximately $6 \times 10^{46}$ different static reward schedules $$\left(\left( {\begin{array}{*{20}{c}} {100} \\ {25} \end{array}} \right)^2\right)$$ consistent with our constraints. A choice engineer that is equipped with an accurate quantitative model of the decision maker can search the optimal reward schedule, the one that maximally biases choices, by optimizing over the different possible schedules in view of her model (see Supplementary Methods). This is, however, not possible for the architect that must rely on qualitative principles to design the reward schedule (as in Fig. 1c). To demonstrate the potency of choice engineering, we assume a decision-maker whose choices follow the QL model^3^. We found in numerical simulations that the bias induced by an engineered schedule (Fig. 1d) is 64% (chance is 50%), substantially larger than the bias induced by the naïve choice architect’s schedule (Fig. 1c), 55% (Fig. 2). Note, however, that effective engineering of behavior requires an accurate model of choice. If the choice engineer is not well-informed and the reward schedule is optimized assuming a different model (Fig. 1e; the CATIE model, see Supplementary Methods), the resultant bias, 53%, can be even smaller than that obtained by the naïve architect (Fig. 2).

## Dynamic reward schedule

The design problem depicted in Fig. 1c–e is static: the full reward schedule is determined in advance and is used for all subjects. This schedule does not take into account heterogeneity between subjects (the fact that different subjects may be characterized by different parameters), as well as the actual sequence of choices (the specific realization of stochastic behavior). A more sophisticated choice engineer can utilize in each trial the subject’s past choices and rewards to dynamically allocate the rewards of the next trial. To test this approach, we trained a deep neural network to maximally bias choice by dynamically allocating the rewards of the next trial, based on past actions and rewards, while complying with the reward schedule constraints (see Supplementary Methods). By definition, a static reward schedule is nested within the dynamic schedule, so if the model of the agent is accurate, the dynamic schedule cannot be worse than the static one. Indeed, we found in our numerical simulations that a QL-optimized dynamic schedule does better than the QL-optimized static one, yielding a bias of 70% for the QL agent. As with the static schedule, a dynamic schedule is effective only if its underlying model of choice is accurate. If optimized for a different model (CATIE) it poorly biases the choices of the QL agent (a bias of 52%, Fig. 2).

## Model comparison

Traditional methods for model evaluation are associated with an estimate of the model’s explained variance. While this approach has proven useful in many studies, it suffers from several shortcomings. First, it is not clear how the complexity of a model—a necessary ingredient in model comparison—should be quantified and utilized^11^. Second, the scope of conditions in which the models are put to test is typically rather limited. Thus, testing the ability of different models to shape behavior (as in Fig. 2) is a novel way of comparing models (see related concept^12^), which is far less restrictive then explained variance in a particular experimental setting. It can be used to compare models of arbitrary complexity—the models are simply compared by their effectiveness in shaping behavior. Moreover, and perhaps more importantly, the competition can compare the potency of quantitative models of choice (choice engineering) to that of qualitative principles (choice architecture) in choice design.

## The competition

The principles underlying operant learning are formally studied in different disciplines, including neuroscience, psychology, economics and computer science. However, one does not need to be formally trained in any of these fields in order to be a good choice designer and participate in the competition. We are all social creatures and have good intuitions as to how to incentivize other humans to make specific choices.

We offer two participation tracks. In the first static track, choice designers are invited to propose static reward schedules in the form of a sequence of rewards (as in Fig. 1). In the second dynamic track, choice designers are challenged to submit a computer program that allocates, in every trial, the reward/s of the next trial, based on the history of choices and rewards. The participants are requested to shortly explain whether their design is based on a quantitative model of choice (making them choice engineers) or qualitative principles and heuristics (making them choice architects). To help choice engineers fine-tune their models, we have already tested 400 subjects using different static schedules that are consistent with the constraints. These datasets are available in the competition website.

## Competition management

The full details of the competition appear in the competition website http://decision-making-lab.com/competition/index.html. In short, the static and dynamic schedules will be independently compared in two separate competitions. The winning schedules are the ones that averaged over the tested sessions, maximize the bias in favor of alternative 1. If possible, each tested subject (an Amazon mechanical Turk worker) will be tested in a single session. To efficiently identify the best schedules, we will use the method of successive rejects^13^ (see Supplementary Methods) that allocates more sessions (more samples) to the so-far better performing schedules. Each participant in the competition is allowed to submit a single static and a single dynamic schedule. Each schedule must be accompanied by a short explanation of the principles or models which guided the design of the submitted application. Should the number of submissions be too large, these explanations will be used for an initial screening of those submitted applications that will enter the competition. The winners of the static and the dynamic schedules competitions will be invited to present their schedules as a talk in a workshop summarizing the competition. If appropriate, they will also be invited to coauthor the paper summarizing the results of the competition. In addition, all the datasets collected during the competition will become publicly available after the competition is concluded.

The deadline for submissions is set to four months from the published date of this commentary.

In conclusion, we believe that the Choice Engineering Competition is a first step in the field of Choice Engineering, providing both a novel way of comparing the potency of different quantitative models to qualitative principles (and to common-sense intuition) and a way of using these quantitative models to shape behavior. In this competition we focus on choices in a specific domain of operant learning—repeated, two-alternative choices with partial feedback. However, this framework can be readily expanded to other operant tasks, e.g., tasks involving multiple-choices, different constraints on the reward schedule, full-feedback, etc. Finally, it can be further generalized to help search for effective learning strategies, from low-level perceptual learning to higher-order skill acquisition.

## Supplementary information

Supplementary Information

## Data Availability

All the code used to generate the results presented in the paper is available upon request from the authors.

## References

[1] Thaler, R. & Ganser, L. *Misbehaving: the Making of Behavioral Economics*. WW Norton, New York (2015).

[2] Thorndike EL (1927). The law of effect. Am. J. Psychol..

[3] Shteingart H, Neiman T, Loewenstein Y (2013). The role of first impression in operant learning. J. Exp. Psychol. Gen..

[4] Mongillo G, Shteingart H, Loewenstein Y (2014). The misbehavior of reinforcement learning. Proc. IEEE.

[5] Shteingart H, Loewenstein Y (2014). Reinforcement learning and human behavior. Curr. Opin. Neurobiol..

[6] Sutton, R. S. & Barto, A. G. *Reinforcement Learning: An Introduction* (MIT Press, Cambridge, 1998).

[7] Erev Ido, Ert Eyal, Roth Alvin E., Haruvy Ernan, Herzog Stefan M., Hau Robin, Hertwig Ralph, Stewart Terrence, West Robert, Lebiere Christian (2010). A choice prediction competition: Choices from experience and from description. Journal of Behavioral Decision Making.

[8] Jolivet Renaud, Schürmann Felix, Berger Thomas K., Naud Richard, Gerstner Wulfram, Roth Arnd (2008). The quantitative single-neuron modeling competition. Biological Cybernetics.

[9] Axelrod R, Hamilton WD (1981). The evolution of cooperation. Science.

[10] Russakovsky O (2015). ImageNet large scale visual recognition challenge. Int. J. Comput. Vis..

[11] Myung IJ (2000). The importance of complexity in model selection. J. Math. Psychol..

[12] Donchin E (1989). The learning strategies project: introductory remarks. Acta Psychol..

[13] Audibert, J. Y. & Bubeck, S. Best arm identification in multi-armed bandits. in *COLT-23th Conference on Learning Theory* 13 p. (2010).

