# Supplementary material for: From choice architecture to choice engineering
Source: Nat Commun. 2019 Jun 26;10:2808. doi: 10.1038/s41467-019-10825-6 (PMC6594951; doi:10.1038/s41467-019-10825-6)
Supplement: Supplementary file 1 — Supplementary Information [file 41467_2019_10825_MOESM1_ESM.pdf]

Supplementary Methods - From choice architecture to choice engineering

Ohad Dan and Yonatan Loewenstein

The Q-Learning Model

The Q-learning model is a reinforcement learning algorithm that has been widely used to model sequential decision-making behavior in humans and animals[1–3]. Applied to this task (formally a one-state MDP), Q-learning describes how the value (expected average reward) associated with each action changes in response to that trial's chosen action and the resultant reward. Formally, at each trial t, the agent selects an action a_t, $a_t \in \{1, 2\}$ and receives a reward r_t, $r_t \in \{0, 1\}$. The updated value of the selected action, $Q_{t+1}(a_t)$, is a weighted average of the previous value $Q_t(a_t)$ and the received reward:

(1) $$Q_{t+1}(a_t) = Q_t(a_t) + \eta\big(r_t - Q_t(a_t)\big)$$

where $0 < \eta \leq 1$ is the learning rate. The value of the nonchosen action remains unchanged, $Q_{t+1}(a \neq a_t) = Q_t(a \neq a_t)$. The difference equation (1) is not complete without specifying the initial conditions. Following experimental findings, we posit that in the first two trials the two alternatives are chosen at random and the initial value of each of the two actions is the reward received the first time this action was chosen[4].

Equation (1) describes how the action values adapt over trials but does not specify how these action values are used to select actions. The mapping between action-values and actions is given by an action-selection rule. Motivated by experimental findings[4], we consider an ϵ-softmax action-selection rule such that:

(2) $$\Pr[a = a_i] = (1 - 2\epsilon)\frac{e^{\beta Q_t(a_i)}}{\sum_a e^{\beta Q_t(a)}} + \epsilon$$

where $0 \leq \epsilon \leq 1$ and $\beta > 0$ are exploration parameters.

Together, Eqs. (1) and (2) fully describe the model. In all simulations, the parameters are: η=0.4, ϵ=0.2, β=257. All three parameters were taken from Shteingart et. al. (2013)[4], where the value of β was scaled such that the product of β and the standard deviation of the difference in rewards associated with the two actions, averaged over all schedules in Shteingart et. al. (2013)[4], is equal to the same product in the current problem.

The CATIE model

The contingent average, trend, inertia, and exploration (CATIE) model is a phenomenological model developed to explain human behavior in tasks of sequential choices with partial feedback, similar to the task of the competition[5]. The model describes an agent which in each trial, chooses according to one of four modes: exploration, exploitation, inertia, and a simple heuristic. In the first two trials, the agent samples the two alternatives in a random order. In each consecutive trial, one of the four modes is selected according to the following probabilistic rules:

1. If the agent chose the same alternative in two consecutive trials and their outcomes differed, it would choose in the subsequent trial the trend (or heuristic) mode (see below) with a probability τ.
2. If the trend mode was not chosen, the agent would choose the explore mode (see below) with a probability $\mathrm{p\_explore}$.
3. If neither trend nor explore modes were chosen, the agent would choose, the inertia mode (see below) with a probability ϕ.
4. If none of the above modes was chosen, the agent would choose the contingent average mode (see below).

In the trend mode, the agent would choose the same alternative as in the previous trial if the trend of last two outcomes was positive, i.e. if the last outcome was greater than the one preceding it. If the trend was negative it would switch to the other alternative:

$$(a_{t+1}|\text{trend\_mode}; a_t, a_{t+1} = a^i) = \begin{cases} a^i & r_t > r_{t-1} \\ a^{\bar{\imath}} & r_t < r_{t-1} \end{cases}$$

Where $a^i, i = \{1, 2\}$ are the available alternatives in the task, a_t is the action at trial t (i.e. the choice of a^1 or a^2) and $r_t \in \{0, 1\}$ is the reward at trial t. In the context of the competition (binary rewards), trends are manifested by a sequence of either reward→ no reward (negative trend), or no reward→ reward (positive trend).

In the explore mode, the agent chooses between the alternatives randomly, with an equal probability. The probability of entering the explore mode is a function of the sequence of surprises experienced by the agent up until the current trial. $\mathrm{Surprise}_t \in [0, 1]$, is calculated at trial t as follows:

$$\mathrm{Surprise}_t = \begin{cases} \dfrac{\left|\mathrm{Exp}_t^i - V_t^i\right|}{\mathrm{ObsSD}^i + \left|\mathrm{Exp}_t^i - V_t^i\right|}, & \text{if } \mathrm{ObsSD}^i > 0 \\ 0, & \text{otherwise} \end{cases}$$

Where Exp_t^i is the reward the agent expects to receive at trial t after choosing alternative i at trial t (the alternative's *contingent average*, see below), V_t^i is the actual reward obtained by choosing alternative i at trial t, and ObsSD^i is the standard deviation of all payoffs observed from alternative i at trials 1 through t. Note that at each trial the surprise is calculated relative to the single alternative chosen by the agent. Given the sequence of previous surprises, the probability of

entering the explore mode, conditioned on not choosing the heuristic mode is:

$$\text{p\_explore} = p(\text{Explore}|\text{No\_Heuristic})_{t+1} = \epsilon * \frac{1 + \text{Surprise}_t + \text{Mean\_Surprise}}{3}$$

Where ϵ is a free parameter capturing a basic exploration tendency and Mean_Surprise is the average surprise in trials 1 through t. Thus, the probability of exploration is minimal (equals $\frac{\epsilon}{3}$) when the surprise is minimal (both Surprise_t and Mean_Surprise equal 0), and maximal (approaches ϵ) when the surprise is large.

The inertia mode simply repeats the same choice of the previous trial.

In the contingent average (CA) mode, the agent chooses the alternative associated with the higher *k*-CA, defined as the average payoff observed in all previous trials which followed the same sequence of *k* outcomes (the same contingency of length *k*). At its initialization, the agent uniformly draws an integer k from the discrete range $\{0, \dots, K\}$ ($k \sim u\{0, K\}$, K is a free parameter). Each time the agent enters the CA mode, it calculates *k*-CA for the two alternatives. For each alternative i, *k*-CA is the average reward obtained in trials which followed a contingency of length k which is identical to that of the most recent k-contingency and were followed by choice of i. In particular, k=0 implies averaging all the outcomes of an alternative. To illustrate this process, consider the following sequence of 10 choices in alternative 1 ("A") and 2 ("B"): [A, A, A, A, A, A, A, B, B, B], implying the agent is currently at the 11th trial, and assume that these choices yielded the following reward sequence [1, 0, 0, 0, 1, 0, 0, 1, 0, 0]. An agent characterized by a k=2 would imply that the current contingency is [0, 0] (the sequence of last two rewards). Previous indices where the same reward sequence was observed are 2, 3 and 6; the choices which followed these sequences are A, A and B respectively (at trials 5, 6 and 9); and the rewards which followed these contingencies are 0, 1 and 1 (the rewards of trials 5, 6 and 9). This would imply that the contingent averages of alternatives A and B are 0.5 and 1 respectively. Hence, in the current trial alternative B, which has the higher contingent average, would be chosen. If for alternative i there are no past *k*-contingencies matching the most recent contingency, the value of a different random *k*-contingency replaces the contingent average for that alternative. A random *k*-contingency value is obtained by considering the set of all reward sequences of length k which were followed by choice of i, randomly choosing one of them with a uniform probability and calculating its contingent average value. Note that choosing uniformly from past trials implies that recurring contingencies have higher probability of being chosen. If there are no contingencies of length k that are followed by a choice of i, a smaller k (*k:=k-1)* is chosen iteratively until at least one contingency exists. Since the model is defined to sample the two alternatives in the first two trials, for *k*=0 it is guaranteed that both alternatives would have a *0*-contingency value and hence that the CA mode is well defined.

As described above, the model is characterized by four free parameters (one for each mode: τ, ϵ, ϕ, K). The parameters of the model were estimated from behavior[5] and these estimations were used in all simulations: $\tau = 0.29$, $\epsilon = 0.30$, $\phi = 0.71$, $K = 2$.

Finding an optimal static reward schedule

The optimization procedure operates iteratively. In the first iteration, an initial sequence S_1 is generated by randomly shuffling, independently for the two alternatives, a sequence that complies with the global sequence constraints (rewards are assigned to 25 of the experiment's 100 trials). In the k'th iteration, a partially reshuffled sequence S'_k is generated from the current sequence S_k by choosing $t \sim u\{2, T_k\}$ indices, with uniform probability without replacement, independently for the two alternatives, and randomly reshuffling the rewards assigned to the trials of these indices. The schedule S'_k is tested N times (N independent agents) and compared to the bias previously obtained for the unshuffled schedule. This procedure is repeated m times and the schedule used for the subsequent iteration is the one that yields the largest bias out of the $m + 1$ tested schedules (m shuffled schedule + the original schedule). The variable T_k, which represents the 'temperature' of the algorithm progressively decreased to zero such that $T_k = \phi(k)$, where $\phi(k) = \left\lceil \frac{A-k}{B} \right\rceil$ where A, B are parameters. The parameters we used for this procedure are $A = 10{,}000, B = 100, N = 500, m = 10$. Finally, we note that the reward of the last trial cannot change behavior. Hence, it is never advantageous to allocate a reward to the last trial of the bias-target alternative. If the stochastic optimization converges to a solution which does not meet this criterion, the reward schedule of the bias-target alternative is modified to conform with this heuristic by reallocating the reward of the last trial to a random trial currently not associated with a reward. Similarly, if a reward is not assigned to the last trial of the anti-bias alternative, one of the rewards associated with that alternative is randomly chosen and reallocated to the last trial.

Note that this method is not guaranteed to find a global optimum, nor to converge to the same solution in independent applications. Therefore, we repeated this procedure $n = 20$ times and chose the schedule that yielded the maximal bias. To avoid selection bias, the bias measured in Fig. 2 was measured by simulating the schedule on 1,000 independent agents.

Finding an optimal dynamic reward schedule

We used an artificial neural network to dynamically allocate rewards to the two alternatives. In each trial, the network receives an input which encodes the history of past choices and rewards (see also below), and its output is the allocation of reward to each of the alternatives in that trial. To guarantee that the constraints are satisfied, the network could not assign

additional rewards to an alternative in which 25 rewards have already been assigned. Similarly, if the number of remaining trials is equal to the number of remaining rewards, a reward is assigned in every trial until the end of the session.

The network is composed of four layers L_0, L_1, L_2, L_3. The activity of neuron i in layer $l > 0$, x_i^l is given by:

$$x_i^l = \tanh\left(\sum_{j=1}^{N^l} w_{ij}^l x_j^{l-1}\right)$$

where N^l is the number of neurons in layer l and w_{ij}^l is the strength of connection from neuron j in layer $l-1$ to neuron i in layer l.

The number of neurons in layers L_1 and L_2 is $N^1 = N^2 = 100$. The output layer is composed of four neurons ($N^3 = 4$). Each of the four neurons denotes a particular allocation of rewards (no rewards, reward only to alternative 1, reward only to alternative 2, rewards to both alternatives) and the allocation of rewards is determined by the identity of the most active neuron in that layer.

The weights were trained iteratively using a genetic algorithm[6]. In each iteration (generation) ten sets of weights were generated. The performance of each of the ten sets of weights was evaluated by estimating the average bias associated with that set of weights, as was done for the static reward, and the two sets that yielded the largest bias were identified and used for the construction of weights for the next iteration. The ten sets of weights were constructed in the following way: one set of weights was chosen from a uniform distribution, $w_{ij}^l \sim u(-0.05, 0.05)$, two more sets of weights were the two best performing sets of the previous generation, denoted as parents of the current generation. Seven more sets of weights were constructed using a crossover procedure, in which each weight is independently drawn from one of the parents' sets of weights. Finally, each of the weights were 'mutated' in a probability of 0.2. A randomly drawn number from a uniform distribution $\Delta w_{ij}^l \sim u(-0.005, 0.005)$, independently drawn for each weight and each set was added to each mutated weight. This procedure was terminated when the improvement in bias, relative to the average of the last 400 generations, was smaller than 0.01. The input layer L_0 depended on the model used. When constructing a dynamic reward schedule for the QL model, the input layer was composed of four neurons whose activity in every trial t denotes (1) trial number; (2, 3) total number of reward allocated in previous trials to alternatives 1 and 2; (4) the difference between the two alternatives' Q-values, $Q_t(a_1) - Q_t(a_2)$, as estimated from previous trials using the true parameters of the model.

When constructing a dynamic reward schedule for the CATIE model, the input layer was composed of six neurons whose activity denotes (1) trial number; (2, 3) total number of reward allocated in previous trials to alternatives 1 and 2; (4) an indication for current *trend* represented by the values: -1 if trend is not applicable (if two previous actions are not identical), 0 if the trend is negative, and 1 if the trend is positive; (5) the probability of using the exploration mode (p_explore for the explore mode); (6) the alternative chosen in last trial (for the inertia mode).

Sorting applications with 'successive-rejects'

Consider a reward schedule submitted to the competition. If tested over different subjects, it is likely to yield a different bias for each subject. This is due to stochasticity in the behavior of the individual subject and the heterogeneity between the subjects. In this sense, the reward schedule defines a distribution of biases over subjects, and our goal is to identify the reward whose distribution is associated with the largest mean. Successive-rejects[7] is a method for an efficient allocation of a finite number of n draws (subjects) from M distributions (schedules) to optimally identify the distribution with the highest mean. Successive-rejects operates iteratively. In the first iteration, all distributions are sampled. In each iteration, the distribution associated with the lowest mean (as estimated from all draws up to this point) is removed from the set of considered distributions and the remaining distributions are reevaluated by drawing a specific number of additional samples each. In order to compute the number of draws used in iteration k for each of the remaining distributions, we denote by n_k the cumulative number of draws from that distribution up to iteration k (included). By definition, $n_0 = 0$ and $n_k - n_{k-1}$ is the number of draws used in iteration k per each remaining distribution. According to the method of successive rejects,

$$n_k = \left\lceil \frac{1}{\overline{\log}(M)} \frac{n-M}{M+1-k} \right\rceil$$

where

$$\overline{\log}(M) \equiv \frac{1}{2} + \sum_{i=2}^{M} \frac{1}{i}$$

The algorithm terminates after $M-1$ iterations, when only a single distribution (schedule) remains.

References

1. Barto, A. G., Sutton, R. S. & Watkins, C. J. C. H. Learning and Sequential Decision Making. in *Learning and Computational Neuroscience* 539–602 (1991).

2. Mongillo, G., Shteingart, H. & Loewenstein, Y. The Misbehavior of Reinforcement Learning. *Proc. IEEE* **102**, 528–541 (2014).

3. Daw, N. D., Gershman, S. J., Seymour, B., Dayan, P. & Dolan, R. J. Model-based influences on humans' choices and striatal prediction errors. *Neuron* **69**, 1204–1215 (2011).

4. Shteingart, H., Neiman, T. & Loewenstein, Y. The role of first impression in operant learning. *J. Exp. Psychol. Gen.* **142**, 476–488 (2013).

5. Plonsky, O. & Erev, I. Learning in settings with partial feedback and the wavy recency effect of rare events. *Cogn. Psychol.* **93**, 18–43 (2017).

6. Miikkulainen, R. *et al.* Evolving Deep Neural Networks. in *Artificial Intelligence in the Age of Neural Networks and Brain Computing* (eds. Kozma, R., Choe, Y., Alippi, C. & Morabito, F. C.) 293–312 (Academic Press, 2018).

7. Audibert, J. Y. & Bubeck, S. Best Arm Identification in Multi-Armed Bandits. in *COLT-23th Conference on learning theory* 13 p. (2010).
